# Sad Bereavement

**Published:** 1872-09

**Authors:** 


Sad Bereavement.—The papers announce to us the sad intelligence that Lieutenant Reid T. Stewart of the Fifth U. S. Cavalry met his death on the 28th of August at the hands of the Indians, near Tuscon, Arizona. Lieutenant Stewart was the son of Dr. J. L. Stewart of Erie Pa. He graduated at West Point in June 1871 and in November of the same year entered upon his duties in Arizona. He was beloved and honored by his comrades in arms by whom his loss will be severely felt. At the time of his death he was but a little more than twenty-one years of age. The sad bereavement of the parents is something too sacred to be spoken of in common words. We extend to them our heart felt sympathy.

